# Effects of health educational and participatory consumer group interventions in improving food handling practices in regional director of health services area Kalutara, Sri Lanka: non-randomized controlled community trial

**DOI:** 10.1186/s12889-024-18481-2

**Published:** 2024-04-06

**Authors:** Lasantha Krishan Hirimuthugoda, Padmal De Silva, Palitha Abeykoon

**Affiliations:** 1 Illinois Institute of Technology, 10 W 35th Street, Chicago, IL 60616-3793 USA; 2 National Institute of Health Sciences, Ministry of Health, Colombo, Sri Lanka; 3 World Health Organization – Country Office, Colombo 05, Sri Lanka

**Keywords:** Food regulations, Food handlers, Food handling practices, Food establishments, Consumer groups, Educational packages

## Abstract

**Introduction:**

Safe and nutritious food is the key to sustaining life and promoting good health. Unsafe food creates a vicious cycle of disease and malnutrition, particularly affecting infants, young children, the elderly, and the sick.

**Methods:**

The study consisted of two phases, a descriptive cross-sectional study, and an intervention study. Both studies were conducted in the Regional Director of Health Services area, Kalutara, Sri Lanka. The descriptive cross-sectional study [food handlers (*n* = 904), food establishments (*n* = 421)] was conducted with the objective of determining factors associated with food handling practices among food handlers and in food establishments. The interventional study was a three-arm non-randomized controlled community trial (*n* = 50 per arm) with interventions of a participatory consumer group, educational package group, and control group.

**Results:**

The food establishments assessment tool (FEAT) contained 11 domains including 75 items with more than a hundred assessment points with a guide to conduct an assessment of food handling. The descriptive cross-sectional study found that food handlers’ knowledge of food handling practices of storing milk, fish, and meat and fast-food items containing fish and meat was very poor (96.6%). Visibility of the last place of processing inside the food establishments to consumers was inadequate (19.2%) and the absence of the above-mentioned factor was significantly associated with an unsatisfactory level of food handling score in food establishments (*p* = 0.03). The unsatisfactory level of food handling was significantly higher among food establishments with non-personal ownership (*p* = 0.005), a low number of notices issued by legal authorities (*p* = 0.02), dereliction of duty by owners/managers on supervising (*p* < 0.001) and lack of medical certification to food handlers (*p* < 0.0001). Participatory consumer group intervention and educational package interventions were effective in improving food handling practices in food establishments and among food handlers (*p* < 0.0001). Two independent sample analysis using the Mann–Whitney U test showed, the best improvement in food handling practices was by participatory consumer group intervention (*p* < 0.0001) and the second was educational package intervention (*p* < 0.0001).

**Conclusions:**

Knowledge and practices of food handling among participants were poor. A participatory consumer group is more effective than an educational package on improving food handling practices both among food handlers and in food establishments.

**Supplementary Information:**

The online version contains supplementary material available at 10.1186/s12889-024-18481-2.

## Introduction

Hazardous food has been a serious public health problem since history was first recorded, and many food safety problems encountered today are not new [1]. Infections and diseases arising from contaminated food remain threats to global public health [2]. An International analysis by the World Health Organization (WHO) revealed in 2019, that an estimated 600 million (almost 1 in 10 persons in the world) fall ill after eating contaminated food, and 420 000 die each year, resulting in the loss of 33 million healthy life years (Disability-Adjusted Life Years). Children under 5 years of age carry 40% of the foodborne disease burden, with 125,000 deaths every year [3]. Diarrhoeal diseases are the most common illnesses resulting from the consumption of contaminated food, with 550 million falling ill with 230 000 deaths every year [4]. Indoor Morbidity and Mortality statistics of Sri Lanka in 2017 revealed that categories (International Classification of Disease 10 coding: A00 – A09), showed the rate of mortality due to infectious intestinal diseases for the 100,000 population from 2010 to 2017 as a plateau (as total in all age groups). In 2017, infectious intestinal diseases prevailed in all age categories with the highest rate among the working age group (18–65 years) [5].

Food safety is of utmost importance in the twenty-first century [6], since unsafe food is a major public health problem in both developed and developing countries. Food quality and safety are the totality of characteristics of the food products that bear on their ability to satisfy all legal, customer, and consumer requirements [7]. Food safety in Sri Lanka is ensured through the Sri Lanka Food Act enacted by the Parliament in 1980 with 2 amendments made in 1991 and 2011, plus 40 Food Regulations which help to streamline and enforce the principal food laws in a comprehensive manner in the country. Food (Hygiene) Regulations of Sri Lanka 2011 and Food (Registration of Premises) regulation should be applied to all establishments dealing with the processing, transport, distribution, handling, storage, or sale of food or any other matters related to food establishments [8, 9].

“Food handler” means any person who directly handles packaged or unpackaged food, food equipment, utensils, or food contact surfaces and is therefore expected to comply with food hygiene requirements. “Food establishment” is defined as any building or area in which food is handled and the surroundings under the control of the same management [9]. Food handling is inevitably involved with food preparation and consumption, and many developing countries operate with manpower for manufacturing, packaging, and distributing. As 97% of food-borne diseases occur due to malpractices of food handlers in food establishments [10]. Any food safety training program for food handlers should include the contents defining awareness and responsibilities, implementing the training program, regular instruction and supervision, and periodic updated refresher training [11, 12]. A review provided evidence of the effectiveness of food handler training programs, in conjunction with certification, to improve the knowledge and practices of food handlers [13].

In Sri Lanka, currently, consumer rights movements are operating on a small scale to fight for consumer rights, but hardly empower consumers individually to strengthen their knowledge and awareness of their rights on their purchases. However, provisions of the Consumer Affairs Authority Act of Sri Lanka clearly state the creation of informed groups of the public as consumer organizations, to promote, assist, and encourage their rights on purchases [10]. In Public Utilities Commission Act of Sri Lanka is defined to protect the interest of all consumers, and consumer groups to be informed and their consensus obtained at the time the commission exercises its power over matters related to industries [14].

According to the statistics, Sri Lanka showed a persistent of mortality of reported infectious intestinal diseases, poor food handling practices by handlers, and the opening of food establishments of low quality in every corner of the country [5]. Furthermore, there is little or no information available on the level of food safety knowledge and factors affecting food handling practices regarding compliance with Sri Lankan laws among food establishments. Also, no information is available on how the effects of packaged training provided to food handlers and measures for empowerment of consumer groups for their own food safety will reciprocally affect food safety in the establishments in Kalutara, Sri Lanka. Therefore, these could hinder the development of appropriate disease prevention and public health intervention strategies. Hence, the present study is designed to assess factors affecting food safety and practices in food establishments toward compliance with Sri Lankan law.

### Methodology

Our objective is to determine the food handling practices of food handlers and food establishments, the factors associated with them, and the effect of health education and participatory consumer group interventions in improving food handling practices among food handlers and food establishments in the Regional Director of Health Services area Kalutara (RDHS), Sri Lanka. This study was conducted in two phases.

1. Phase 1: Assessment of factors associated with food handling practices among food handlers and food establishments (Descriptive cross-sectional study).
2. Phase 2: Evaluation of effectiveness of an educational package and participatory consumer group interventions to improve the food handling practices among food handlers and food establishments (Non-randomized controlled community trial).

**Fig. 1:**
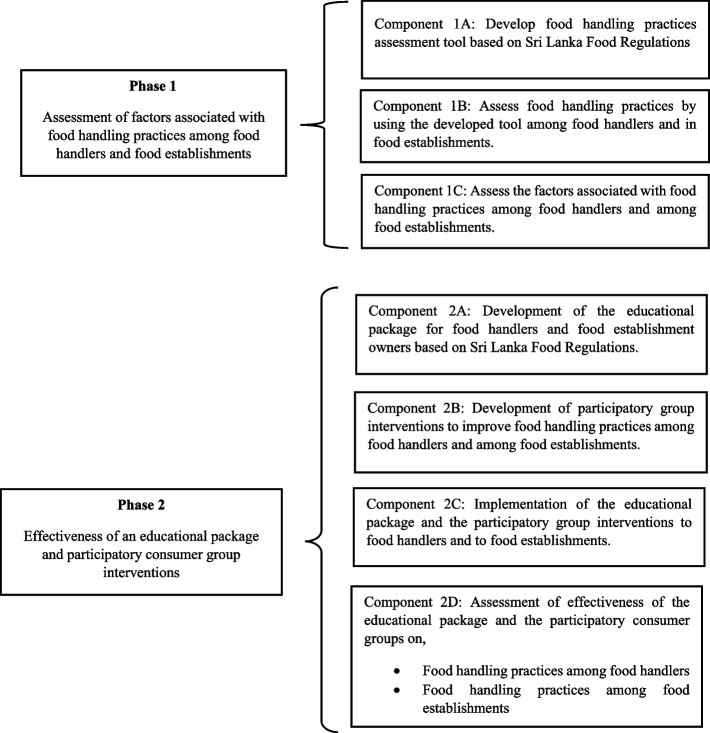
Schematic presentation of phase 1 and phase 2 and components

**Fig. 2:**
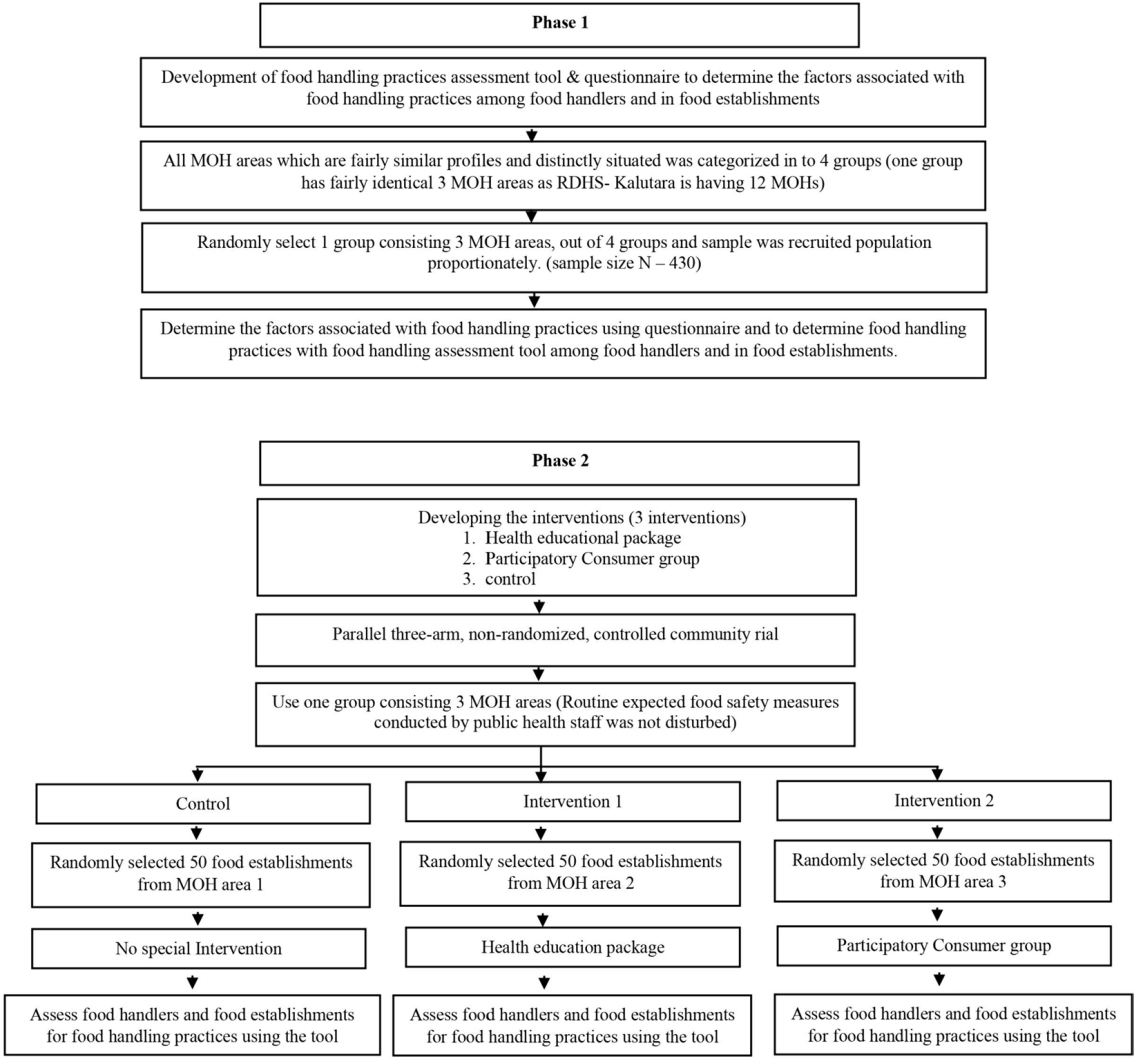
Flow chart of the study

Phase 1 has three components and Phase 2 has four components. The different components of the two phases are summarized in Figs. 1 & 2.

In phase 1, the development of food establishments’ assessment tool (FEAT) was carried out to mark inspection scores for food establishments in accordance with Hygiene Regulation 1742/ 26 of Sri Lanka Food Regulations and several other reviewed international food safety protocols (Supplementary file 1). The questionnaire had components on the following areas of socio-demographic factors, factors associated with food handling practices among food handlers and food establishments (Knowledge, education, sex, chain of food premises, group / single ownership… etc.), and factors associated with management of food establishments ensuring food safety. FEAT was attached as Supplementary file 2.

In phase 2, an educational package was designed targeting food handling practices among food handlers and owners of food establishments in the Kalutara RDHS area, and the intervention was implemented to the target group by the Medical officer of health. The intervention was designed mainly based on, Sri Lanka Food Regulations, findings of the descriptive study, from a review of literature on methods of changing food handling practices through educating of food handlers, and supervision and guidance of project supervisors, and consisted of a workshop, posters displayed at food establishments, distribution of leaflets and weekly refresher training to food handlers. The Most of critical points identified from the descriptive phase of the study were addressed specifically on facts of storing food items, receiving medical certifications, and providing timely and refresher training to food handlers. All food handlers and owners were exposed to practical sessions on storing food items in refrigerators and hand washing practices following the PowerPoint presentation about all aspects of food handling practices. Weekly visits to food establishments were included with replacing the displayed posters with new one, distributing leaflets, and an onsite short-refresher training. Participatory consumer group intervention had four components, which include the formation of consumer groups for each Public Health Inspectors’ (PHI) area, the development of a consumer rating tool to rate food establishments, the development of a Standard Operating Procedure (SOP) for consumer groups to use at the time of operation and development of evaluation form for owners of food establishments to review consumer groups operations at their premises. With the discussion among the Supervisory Public Health Inspector (SPHID), Supervisors, RDHS –Kalutara, Medical officer—Food and Drug (MO—F&D), and Food and Drug Inspector (F&DI), it was suggested, that consumers should be empowered with legal components and facts of the Food Act and hygiene regulation after they were organized as consumer groups. Ten consumers were selected for each group and similar types of groups were formed in all four PHII areas of the Bulathsinhala Medical Officer of Health (MOH). Groups were educated on Sri Lanka Food Regulations, developed SOP, and work they had to carry out with consumer rating tools. Consumers, who own any type of business, especially food-related businesses, and who are working in the Department of Health, departments related to forces or civil protection were excluded.

The consumer rating tool is a self-inspection list (Supplementary file 3) filled by a consumer after consuming food from the respective food establishment. This tool has rating items for the general condition of the establishment, serving and processing area, personnel hygiene of the food handlers, other facilities like clean toilets for consumers and waste disposal, and responsibility of the owners/managers over supervising food handlers. Consumers marked the rating as “Satisfactory, Needs Improvement, and Unsatisfactory”.

Consumer groups had to visit selected food establishments in their area and mark the level of hygienity of the food establishment using the developed consumer rating tool on food establishments. Feedback was collected from the food establishment owners to assess the operation and assessment of consumer groups at the time of their intervention. Intervention 1 is a developed educational package consisting of conducting workshops, displaying posters at food establishments, refresher training, and handing over leaflets to improve the food handling practices of the food handlers. Intervention 2 is a developed participatory consumer group that operates at food establishments to improve food handling practices among food handlers. Control is the area, where no interventions were planned to conduct.

### Study setting

A randomly selected sample of food establishments and food handlers in the RDHS area, Kalutara 2018 were considered as the target population. Kalutara district was selected as it represents all races, ethnicities, and levels of economic status equally to represent all other districts of the country. The following three sources of databases were used to compile the final sampling frame of food establishments in each divisional secretary area by using field-level food business registration data, register of business registration, and list of tax-paying data for all business institutes/places.

Out of the 12 MOH areas under the RDHS/Kalutara, all MOH areas with similar profiles and distinctly situated were grouped for the study, consisting of three MOH areas to one group for preventing contamination in the intervention component [15]. If the food establishment selected was not opened at the time, a repeat visit was made within the other days (most probably the next day), in which data collection was conducted in a particular MOH field. A maximum number of 3 food handlers (Kitchen—1, working as manager or owner of the food premises—1, working other areas of the premises—1) was recruited randomly.

The selection criteria is to include licensed food establishments that were opened during the daytime (The feasibility of the study is constrained to do data collection at night. Most of the licensed food establishments were limited only to the daytime, or else to open both day and nighttime. Less number of establishments are found to open only at night). The study excluded food establishment owners and food handlers, who were unable to hear due to hearing defects during the time of data collection [16], and food handlers who had been working in the selected food establishments for less than 3 months. FEAT and developed interviewer-administrated questionnaires were employed to determine the level of good food handling practices and the factors associated with food handling practices among food handlers and in food establishments. Data collection was carried out by one team comprising of principal investigator (PI) and a team of field investigators from August 2018 – November 2018. A practical session was carried out prior to the data collection, in an area that was not included in the study proper.

The educational package was implemented as a community-based controlled, non-randomized trial in the Walallawita MOH area. The selection of food establishments was done by using the sampling frame and the implementation of the package was conducted as day sessions, displaying of posters, distribution of info-sheets, and refresher sessions at their establishments.

**Fig. 3:**
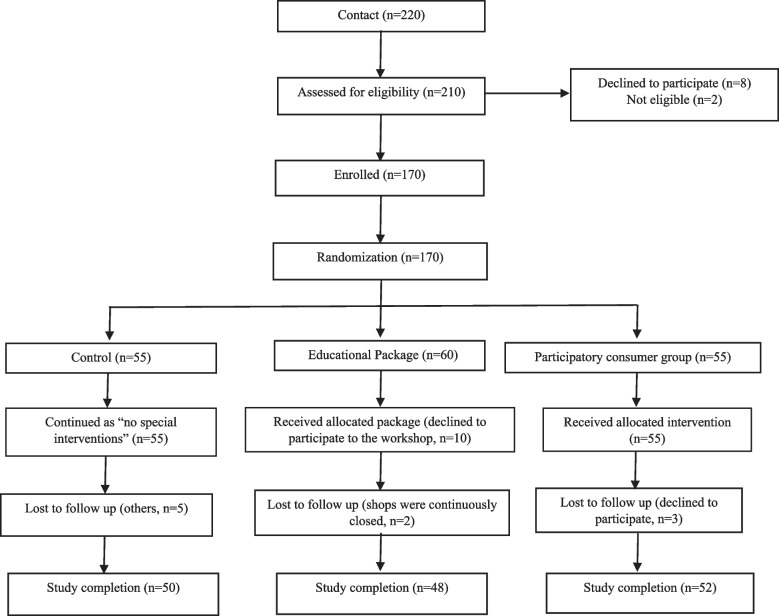
Consort flow diagram of the study

Consumer groups gathered, acted, and dissolved according to SOP and the meeting was conducted before going to visit the food establishments. Monthly visits were conducted for four months as four groups to designated PHI areas of the Bulathsinhala MOH area. At the end of every visit, groups conveyed the rating scores and rating levels of the food establishments to the owner/ manager of the food establishments. All documented ratings were handed over to the PI at the meeting of the MOH office every month. Selected food establishments were enrolled for six months duration for the conduction of the study and post-assessment. Feedback was distributed among owners of food establishments, which were selected to intervene with participatory consumer groups. Owners/managers were instructed not to convey their evaluation to consumer groups. Owners were informed about the intervention prior to initiation. After the consumer group operation at their premises, owners filled out the evaluation form and handed it over to PI. No interventions were conducted in one MOH area named Bandaragama. Consort diagram to show the flow of the study was included in Fig. 3.

The developed FEAT was employed to conduct the assessment after one month of both interventions in all three MOH areas. The inspection score was given to each food establishment and the final value was re-calculated as average. The difference in inspection scores before and after the interventions was analyzed among three groups. Using the same sample frame, the sample size was calculated as 50 per arm [17].

Before the data collection, the study was designed to reduce the major forms of bias and confounders to preserve internal validity. Assigning sample size for both phases of the study (*n* = 904, *n* = 421, and *n* = 50), using random sampling method selecting the food establishments and food handlers for both phases, training all data collectors, testing pilot work in different study proper and using interviewer-administered questionnaires served the internal validity of the study. After data collection, 10% of the data collection was reviewed and cross-checked by a set of independent experts from the National Institute of Health Sciences (NIHS), Kalutara. All the data was manually checked and entered into Excel sheets. A study was conducted in the most representative district of Sri Lanka in aspects of socio-demographic and economic variables. To secure the external validity, the sample was selected to represent the whole district.

Data analysis was done by using the SPSS-21 package. For all outcomes, the chi-square test was used to assess the association between food handling factors over the score of food handling practices, and 5% significance and 95% CIs were reported. The data distribution for phase two of the study (interventional study) was formally assessed and with evidence for departure from normality was found, non-parametric techniques were used with no adjustment. Inspection score reported non–normal distribution in visual methods by histograms, Q – Q plots, skewness of the distribution at ± 1.96 (*p* < 0.05), Kolmogorov–Smirnov test with significance of *p* = 0.05 and a Shapiro- Wilk test with significance of *p* < 0.0001. Since the three groups are independent, the Kruskal–Wallis test was used to assess any significant difference among the three intervention groups pre and post-intervention separately using mean ranks of inspection score [18]. Mann–Whitney test was used to assess the significance difference between the two groups separately using mean ranks of inspection scores to identify which group showed the significance [19].

## Results

The study enrolled 421 food establishments and 904 food handlers in the descriptive component of the study and 50 food establishments per arm in a three-arm controlled community trial.

Socio-demographic characteristics, training, and medical certification of food handlers were reported in Table 1.

**Table 1:** Distribution of socio-demographic factors, training received and medical certifications among food handlers

Description	n	%
Gender		
Female	370	41.0
Male	534	59.0
Ethnicity		
Sinhala	849	93.9
Tamil	29	3.2
Burger	3	0.3
Moor	23	2.5
Religion		
Buddhist	850	94
Catholic	3	3
Hindu	25	2
Islam	26	2.9
Age Category^a^		
Teenage (Less than 20 yrs)	25	2
20—29yrs	119	13.2
30—39yrs	172	19
40 -49yrs	251	27.8
50—59yrs	180	19.9
60 and above	157	17.4
Marital Status		
Married	778	86.1
Unmarried	124	13.7
Widowed	2	2
Divorced	0	0
Others	0	0
Income level in LKR		
Less than 10,000	180	19.9
10,001–30,000	358	39.6
30,001–50,000	235	26
50,001–100,000	100	11.1
> 100,000	24	2.7
missing	7	0.8
Level of education		
No schooling	18	2
Up to grade 5	207	22.9
Up to Ordinary Level (O/L)	422	46.7
Up to Advanced level (A/L)	227	25.1
Holding a diploma or degree	30	3.3
Undergone any type of formal training as a food handler		
Yes	64	7.1
No	840	92.9
Undergone any type of non-formal type training as a food handler		
Yes	241	26.7
No	663	73.4
Undergone any type of formal or non-formal training as a food handler		
Yes	305	33.7
No	599	66.3
Mode of any type of training received^b^		
From MOH office	7	2.3
By Business related training sessions	5	1.6
From training at Hotel school	47	15.4
At the time, obtaining NVQ certification	9	3
By experience	230	75.4
By parents and Family	7	2.3
Years of experience		
Less than 1 year	209	23.1
From 1 – less than 2 years	145	16
From 2 – less than 5 years	185	20.5
From 5 – less than 10 years	126	13.9
From 10 years onwards	239	26.4
Availability of medical certification to work as a food handler		
Yes	79	8.7
No	825	91.3

^a^Average age 45.7 years, Mode 45 years

^b^Among 305 food handlers who received any form of training (response “Yes” to the previous question)

The majority of participants were Sinhala Buddhists. Food handlers were equally distributed among all age categories with the highest percentage between 40–49 and 62% below 50 years of age. More than 86% of the food handlers in the sample were married and almost 60% of them were having monthly income of less than 30,000 LKR. Almost 47% of them were only educated up to Ordinary Level. As reported by literature, formal training was the optimal training, that should be provided to food handlers, though 92.9% of the sample had not. 75.4% of food handlers are undertaking their job by experience while 15.4% received training from the hotel school. Only 2.3% had received routine training given by medical officer of health to food handlers.

All three MOH areas scored a maximum score of 5 for the domain listed as water supply and storage and a minimum score of 1 for the domains of availability of medical certification and responsibility of owners of food establishment over optimal practicing of good food handling practices in the premises (Table 2). In addition, taking precautionary measures was good, but maintenance of the processing area and installation of overhead structures and fitting were poor in food establishments.

**Table 2:** Distribution of median values of inspection score calculated in food establishments and in food handlers according to each component of check-list over MOH areas^a^

Domain area	Number of assessment items	Inspection score in Bandaragama (Median/IQR)	Inspection score in Bulathsinhala (Median/IQR)	Inspection score in Walallawita (Median/IQR)	Inspection score in all MOH areas (Median/IQR)
Location and construction of food establishments	9	3.86(0.92)	3.25(0.9)	3.22(0.92)	3.44(0.98)
Maintenance of processing area	14	3.16(0.96)	2.57(0.89)	2.71(0.89)	2.82(0.90)
Display and serving	9	3.83(1.07)	3.27(0.91)	3.00(0.95)	3.37(0.91)
Installation of overhead structures and fittings	3	4.00(0.98)	1.67(0.92)	1.00(0.85)	2.33(0.90)
Water supply and storage	2	5.00(0.64)	5.00(0.43)	5.00(0.36)	5.00(0.55)
Equipment and Utensils	6	4.33(0.91)	4.00(0.98)	3.33(0.99)	4.00(0.95)
Other facilities available	13	3.83(1.08)	3.75(0.96)	3.38(1.01)	3.66(0.97)
Availability of Medical certification	3	1.00(1.01)	1.00(1.04)	1.00(1.09)	1.00(1.04)
Personal hygiene of food handlers	8	3.50(3.29)	3.00(3.05)	2.87(2.80)	3.12(3.08)
Precautionary measures taken	6	4.67(4.48)	4.67(4.24)	3.83(3.83)	4.50(4.23)
Responsibilities of the owners of food establishments	2	1.00(1.68)	1.00(1.33)	1.00(1.68)	1.00(1.56)
Total	75				

^a^all normality tests are non-normal and inspection scores are not normally distributed

Table 3 describes the level of hygienity in food establishments over three MOH areas. Categorization of scoring was done as 1 -2 as “very poor”, 2.1–3 as “unsatisfactory”, 3.1–4 as “satisfactory” and 4.1–5 as “Good” (The establishments that were identified as “Very poor” and “unsatisfactory” were finally included as “unsatisfactory” in the final category. The remaining food establishments that were “Satisfactory” and “Good” were added to the final category of “satisfactory”). 54% of food handlers reported having a satisfactory level of food handling.

**Table 3:** Distribution of hygienity levels according to MOH area

	**Bandaragama**		**Bulathsinhala**		**Walallawita**		**Total**
	n	%	n	%	n	%	n (%)
Very poor	3	1.8	7	4.7	12	11.2	22(5.2)
Unsatisfactory	55	32.7	65	44	52	49.5	172(40.8)
Satisfactory	76	45.2	66	44.6	36	34.3	178(42.3)
Good	34	20.3	10	6.7	5	5	49(11.7)
**Total**	**168**	**100**	**148**	**100**	**105**	**100**	**421(100)**

Out of the study sample, more than 75% of food handlers had a good awareness of General knowledge of food handling activities refrained at food establishments, activities adopted at the time of serving, and consequences due to foodborne disease outbreaks (Table 4). Food handlers’ knowledge of food handling practices of storing milk, fish, and meat and fast-food items containing fish and meat was very poor (96.6%), once it was considered separately from the particular domain, and 8.6% of food handlers had good knowledge of foodborne diseases. In the questionnaire, there were 5 main domains, and out of 5, one domain partly consisted of assessing the knowledge related to milk, fish, and meat. Finally, we assessed the overall knowledge considering all five knowledge domains (Supplementary file 4).

**Table 4:** Distribution of individual knowledge domains according to level of knowledge among food handler

	General Knowledge	Knowledge on activities refrained at food establishment	Knowledge on activities adopted at the time serving food	Knowledge on food-borne diseases	Consequences due to food-borne diseases	**Overall knowledge**
	n (%)	n (%)	n (%)	n (%)	n (%)	n (%)
Very poor	32 (3.5)	38 (4.2)	55 (6.1%)	89 (9.8)	59 (6.5)	31 (3.4)
Poor	4 (0.4)	8 (0.9)	18 (2)	98 (10.8)	36 (4)	6 (0.7)
Satisfactory	0 (0)	110 (12.2)	0 (0)	639 (70.7)	33 (3.7)	53 (5.9)
Good	868 (96)	748 (82.7)	831 (91.9)	78 (8.6)	776 (85.8)	814 (90)
Total	904 (100)	904 (100)	904 (100)	904 (100)	904 (100)	904 (100)

The educational level of food handlers was significantly associated with their non-participation in formal training (*p* < 0.0001). The years of experience in food handling less than 5 years were significantly associated with not undergoing formal training over food handlers who had more than 5 years of experience (*p* = 0.002). The food handlers with < 5 years of experience showed a 2.23 times increase compared to the food handlers who are experienced with > 5 years of food handling.

According to Table 5, owners of food establishments, who were not responsible for supervising and training their food handlers had 13.6 times significantly higher unsatisfactory levels of inspection score among food handlers over to the owners, who were responsible for supervising and training their food handlers (*p* < 0.001).

**Table 5:** Association of selected factors of food handling with inspection score in food handlers (binary form)

Selected factors	Inspection score in food handler		Total (n)	OR (95% CI)	Significance
	Unsatisfactory	Satisfactory			
	n (%)	n (%)			
Level of knowledge*					χ^2^ = 1.02
Satisfactory	442 (52.5%)	425 (47.5%)	867	0.71 (0.36 –1.38)	df = 1
Unsatisfactory	22 (75%)	15 (25%)	37	1.0	*p* = 0.312
Responsibility of owner to supervise and train					χ^2^ = 98.35
No	453 (59.2%)	337 (40.8%)	790	**13.6 (7.2–25.7)**	df = 1
Yes	11 (9.4%)	103 (90.6%)	114	1.0	***p* < 0.001**
Occupying other job while working as a food handler					χ^2^ = 0.01
Employed	86 (51.9%)	75 (48.1%)	161	0.98 (0.7 –1.38)	df = 1
Unemployed	378 (52.6%)	365 (47.4%)	743	1.0	*p* = 0.92
Experience in food handling					χ^2^ = 0.17
> 5 years	184 (50.4%)	181 (49.6%)	365	0.94 (0.72 – 1.23)	df = 1
≤5 years	273 (53.1%)	259 (46.9%)	532	1.0	*p* = 0.68
Missing	7		7		
Medical certification					χ^2^ = 29.1
Not available	445 (53.9%)	380 (46.1%)	825	**3.97 (2.33 – 6.78)**	df = 1
Available	19 (24%)	60 (76%)	79	1.0	***p* < 0.0001**
**Total**	**464**	**440**	**904**		

Non-personal ownerships of food establishments (Directors’ board, family, or government), not having a history of notices and non-visibility of the last place of processing at food establishment were significantly associated with unsatisfactory levels of food establishments compared to their counterparts (Table 6).

**Table 6:** Association of selected factors of food handling related to food establishments with binary form of inspection score of food establishments

Selected factors	Inspection score in Food establishments		Total (n)	OR (95% CI)	Significance
	Unsatisfactory	Satisfactory			
	n (%)	n (%)			
Conducting other food establishments					χ^2^ = 0.44
Yes	11 (35.7%)	21 (64.3%)	32	0.77 (0.36 –1.65)	df = 1
No	157 (40.5%)	232 (59.5%)	389	1.0	*p* = 0.51
Ownership					χ^2^ = 7.67
Not personal	10 (86%)	3 (14%)	13	**5.27 (1.42 –19.5)**	df = 1
Personal	158 (38.6%)	250 (61.4%)	408	1.0	***p* = 0.005**
History of sanctioning					χ^2^ = 1.62
Yes	14 (34.5%)	31 (65.5%)	45	0.65 (0.33- 1.26)	df = 1
No	154 (41.2%)	222 (58.8%)	376	1.0	*p* = 0.2
History of giving notices					χ^2^ = 5.14
No	160 (41.3%)	226 (58.7%)	386	**2.5 (1.11- 5.65)**	df = 1
Yes	8 (23.1%)	27 (86.9%)	35	1.0	***p* = 0.02**
Visibility of last place of processing					χ^2^ = 4.42
No	144 (42.7%)	196 (57.3%)	340	**1.7 (1.03- 2.94)**	df = 1
Yes	24 (29.8%)	57 (70.2%)	81	1.0	***p* = 0.03**
Nature of Food business					χ^2^ = 0.95
Individual, single business	65 (37.2%)	110 (62.8%)	175	0.82 (0.55 – 1.22)	df = 1
All others	103 (42.9%)	143 (57.1%)	246	1.0	*p* = 0.32
**Total**	**168**	**253**	**421**		

Test statistics showed mean ranks of inspection scores among the three study groups (One study group consisted of 50 food establishments) at the beginning of the intervention were not significant. Combined inspection score, inspection score of food establishments, and inspection score of food handlers reported *p* values as *p* = 0.081, *p* = 0.218, and *p* = 0.353 respectively (Table 7).

**Table 7:** Comparison of combined and individual categories’ inspection scores at the beginning of the study in three study groups and combined and individual categories’ inspection score before and after the interventions for the total study samples

Inspection score in three study groups at beginning of the intervention	Educational package	Participatory consumer	Control	Independent samples Kruskal – Wallis test value* P value
	Mean Rank	Mean Rank	Mean Rank	
Combined inspection score in Food establishments and food handlers	64.92	77.46	84.12	5.035 *p* = 0.081
Inspection score of food establishments only	66.98	78.00	81.52	3.049 *p* = 0.218
Inspection score of food handlers only	62.88	80.05	83.57	6.493 *p* = 0.353
Combined inspection score*	**Number**	**Median**	**Quartile**	
			**Q1**	**Q3**
Before the intervention	150	3.170	2.726	3.686
After the intervention	150	3.378	2.774	3.878
Inspection score in food establishments only*		**Median**	**Quartile**	
			**Q1**	**Q3**
Before the intervention	150	3.294	2.612	3.907
After the intervention	150	3.500	3.009	4.146
Inspection score among food handlers only*		**Median**	**Quartile**	
			**Q1**	**Q3**
Before the intervention	333	3.192	2.600	3.608
After the intervention	333	3.325	2.721	3.595

^*^Non parametric test

^*^Inspection score was calculated for food establishments (150) and food handlers (333) separately, then calculated for both as combined score. Final marks were allocated out of 5

Before the intervention, the median of combined inspection scores in both food establishments and food handlers was 3.170, and quartiles were Q1; 2.726, Q2; 3.170, Q3; 3.686. After the intervention, the median time was increased to 3.378 and quartile ranges were Q1; 2.774, Q2; 3.378, Q3; 3.878. Comparing the inspection scores separately to food establishments and food handlers before and after the interventions, the median inspection score was improved from 3.294 to 3.500 for food establishments and from 3.192 to 3.325 for food handlers.

The Kruskal–Wallis test was used to assess significance among three groups of 50 food establishments in each group (a total of 150 food establishments in three groups—two intervention groups and one control group). The null hypothesis of the Kruskal–Wallis test is that the mean ranks of the groups are the same [18]. Mean ranks of difference in combined inspection score in both food establishments and food handlers, inspection score of food establishments, and inspection score of food handlers were significantly different at *p* < 0.0001 among the three populations (Table 8).

**Table 8:** Comparison of mean rank in median difference of combined and individual categories before and after the interventions, over two intervention groups and control at the end of the study

Difference of inspection score before & after the intervention study	Educational package	Participatory consumer group	Control	Independent samples Kruskal – Wallis test value* p value
	Mean Rank	Mean Rank	Mean Rank	
Difference of combined inspection score in both Food establishments and food handlers	76.72	96.96	48.79	31.144 ***p* < 0.0001**
Difference of inspection score of food establishments only	72.73	96.44	57.7	20.913 ***p* < 0.0001**
Difference of inspection score of food handlers only	75.48	96.45	54.57	23.275 ***p* < 0.0001**

With reference to Table 9, Mann – Whitney U was used to determine if there were statistically significant differences between the two groups (of 50 food establishments in each group, a total of 150 food establishments in three groups—two intervention groups and one control group), which were independent and non-normally distributed [19, 20]. The test was applied to the difference(Δ) between combined inspection scores, a difference(Δ) in inspection scores in food establishments, and the difference(Δ) in inspection scores in food handlers.

**Table 9:** Comparison of sample – sample inferential statistics over the difference of inspection score by two independent samples non-parametric test

Difference of combined, food establishments and food handlers inspection score before and after the interventions in two independent samples of 50 food establishments	Mean Rank – Mean Rank	Mann–Whitney	Z—statistics	Independent sample Mann Whitney U Test^a^ – *P* value
**Difference of combined inspection score before & after the intervention in 50 food establishments**				
*Control-Intervention 1*	37.65 – 60.88	631	-4.048	*p* < 0.0001
*Control-Intervention 2*	35.65 – 62.8	535	-4.728	*p* < 0.0001
*Intervention 1- Intervention 2*^b^	41.34 – 59.66	792	-3.159	*p* < 0.002
**Difference of inspection score of food establishments before & after the intervention in 50 food establishments**				
*Control-Intervention 1*	44.12 – 56.88	931	-2.299	*p* = 0.021
*Control-Intervention 2*	39.08 – 61.92	679	-4.024	*p* < 0.0001
*Intervention 1- Intervention 2*	40.98 – 60.02	774	-3.207	*p* = 0.001
**Difference of inspection score of food handlers before & after the intervention in 50 food establishments**				
*Control-Intervention 1*	42.58 – 58.42	854	-2.730	*p* = 0.006
*Control-Intervention 2*	37.49 – 63.51	599.5	-4.407	*p* < 0.0001
*Intervention 1- Intervention 2*	42.56 – 58.44	853	-2.744	*p* = 0.006

^a^non parametric value

^b^Intervention 1: Educational package, Intervention 2: Participatory consumer groups

The difference in combined inspection scores in two study groups of the control-educational package, control-participatory consumer group, and educational package-participatory consumer group demonstrated significant differences between groups as *p* < 0.0001, *p* < 0.001, and *p* < 0.002 respectively. The difference in inspection scores in food establishments and food handlers reported significant associations among all groups.

## Discussion

The effectiveness of the participatory consumer groups was assessed by comparing all three groups together and two groups separately over the improvement of food safety in food establishments. It showed participatory consumer group intervention improved overall food handling practices in the inspection score of food establishments and food handlers. The increase in overall food handling practices was significantly higher over the control group and educational package. In addition, this type of similar effect is shown in food handling practices of food establishments and food handlers individually. Further, participatory consumer group intervention was more effective than educational package significantly. It is pivotal to clarify the law about the rights of consumers and to keep consumers educated on their rights so that they can demand and rely on them [21].

The findings of this community trial reveal the most challenging, but significant realistic benefits in the field of food safety, by introducing follow-up training, empowering consumers, and developing proper assessment tools. Addressing food handling at the community practices in food establishments has been an important starting point in getting this public health issue recognized and on the broader health priority agenda. However, we now follow to move beyond this intervention to consider it at the policy level. Non-participation is one of the most common sources of bias in community-based prevalence studies. The use of Medical Officers’ of Health (MOOH) as the field guides, being conducted by the principal investigator himself and repeated visits and mop-up activities reduced the non-participation of subjects. Having minimal or no intimidating questions, the interviewer-administered questionnaire was used for obtaining socio-demographic information and costing information. A similar method was used to increase participation in the study of De Silva et al., [22]. FEAT was transferred to a mobile app with non-skipping to points to reduce missing data, which was utilized as a strategy by Fernandopulle et al. [23].

The majority of the participants are Male, Sinhala, and Buddhist, as the sociocultural context and the workforce of the country represented in the sample. Our study evaluated the availability and frequency of medical examinations related to food handling, which depended on the epidemiology of food-related diseases in the area. In many contexts, if disease prevalence is very high, the frequency of medical certification to food handlers must be conducted more frequently, which would be decided by the regional epidemiologist of the district. If not, as usual, medical certification should be conducted annually. Satisfactory level of food handling was reported with significance in premises owned to single ownership, being noticed by authorities and visibility of last place of processing to owners/ customers, food handlers having medical certifications, and in food handlers who were supervised and trained by owners. It was agreed with Knife et al., that most of the establishments that had been inspected by legal authorities were more likely to comply with accepted hygienic practices, so did with privately owned businesses [OR = 2.93,95% CI: 1.68, 5.18 and OR = 3.56, 95% CI: 1.81, 7.14, respectively] [24]. Non-compliance among owners/managers to train and supervise food handlers was 87.4% in our study, which agreed with the study of Phillip Seamens [25]. Our study found, that owners who trained and supervised their food handlers responsibly continued significantly higher satisfactory status of hygiene than owners who did not [13.6 (7.2–25.7)].

The training on food safety and hygiene in this study was found to improve the practice of food safety and hygiene among food establishments and among food handlers. Similar studies with educational packages conducted in Turkey and Malaysia as pre-post assessment showed an overall improvement of mean practice score post-intervention in agreement with the findings of this study [26]. Discordant to our study, studies done in Korea and Imo state Nigeria reported no significant change in the practices of food handlers after training [27, 28].

Rating of the services and products represented a key method of assessment of consumer perceived satisfaction. Verbal rating, pencil-and-pen rating, online rating, and telephone surveys were used in many studies on consumer assessments. The rating method of this study was in agreement with the five-star rating method analyzed in the hospital consumer system by Mayo Clinic, USA [29], but discordant with the verbal rating method of study done among Japanese consumers [30]. Both methods demonstrated improved outcomes of the system which was rated by consumers.

Sanctioning over food establishments was lower than the notices given by legal authorities in clear difference. It was agreed with Knife et al., that most of the establishments that had been inspected by legal authorities were more likely to comply with accepted hygienic practices, so did with privately owned businesses [OR = 2.93,95% CI: 1.68, 5.18 and OR = 3.56, 95% CI: 1.81, 7.14, respectively] [24]. Food establishments that were owned by personal individuals showed a statistically significant satisfactory level of hygienity over food establishments owned by families, director boards, or governments, which was concordant with an Odd ratio of 3.41 [1.91–5.51] study done in the USA [31]. That might be due to personal concerns over their personal business than other categories of ownership. Food establishments with food handlers certified medically reported significantly higher satisfactory levels of food handling than the other portions. Implying proper certification for food handlers might positively affect an adaptation of a good level of food handling with the agreement of recommendation by the study of Subaskaran et al. 2009 [32]. Non-compliance among owners/managers to train and supervise food handlers was 87.4% in our study, which agreed with the study of Phillip Seamens [25].

### Strengths and limitations

The study synchronized its findings with the strength of randomly selected 904 food handlers, who were interviewed using interviewer-administered questionnaires, and 421 food establishments, which were assessed by a tool developed with proper methodology. Street food vendors might be the worst sample of socio-demographic and economic status and might be different in relation to food handling practices over food establishments. Those vendors were not reflected in the study. Vendors were not considered with the limits at evaluation of the intervention phase, and considering the effect of non-participation.

Implementation of intervention through the general public with legal components may have encountered several limitations such as: overreacting in offenses at food establishments and misinterpretation of advice as given in SOP. Yet will not have a large effect on the results.

## Conclusions

The study found general knowledge of storing milk, meat, and fish at the correct temperature and storing fast-food items containing fish and meat was very poor among food handlers (2.8%). Factors related to food establishments like non-personnel ownership of food establishments, not being issued with notices by legal authorities on food handling practices of food establishments, and non-visibility of the final place of processing in food establishments were associated with the unhygienic level of food handling practices at food establishments.

Developed and pretested educational package and participatory consumer groups were interventions to improve food handling practices in food establishments. Participatory consumer group intervention (*p* < 0.0001) and educational package interventions (*p* < 0.0001) were effective in improving food handling practices in food establishments and among food handlers. Comparing the interventions only, the participatory consumer group was found to be more effective than the educational package in improving food handling practices among food handlers and in food establishments.

### Supplementary Information

**Supplementary Material 1.**

**Supplementary Material 2.**

**Supplementary Material 3.**

**Supplementary Material 4.**

## Data Availability

All the data generated or analyzed during the current study are available from the corresponding author upon reasonable request.
