# Supplementary material for: Effects of health educational and participatory consumer group interventions in improving food handling practices in regional director of health services area Kalutara, Sri Lanka: non-randomized controlled community trial
Source: BMC Public Health. 2024 Apr 6;24:972. doi: 10.1186/s12889-024-18481-2 (PMC10998395; doi:10.1186/s12889-024-18481-2)
Supplement: Supplementary file 1 — Supplementary Material 1. [file 12889_2024_18481_MOESM1_ESM.docx]

- Sri Lanka Food act, No 26 of 1980(1)
- Hygiene regulation, No1742/26 (2)
- FDA Good Manufacturing Practices (GMP) Checklist for human food (3)
- HACCP principals (4)
- Public health Inspectors guide to food premises inspections in Canada (5)
- a checklist based on current legislation by the food surveillance team of the Municipal Health Secretariat of Porto Alegre, Brazil
- An observational checklist adapted from the “WHO essential requirement for the safety of street-vended foods” in Nigeria (6)
- 57-item checklist based on the 1976 federal food service sanitation manual in Florida (7)

Reference:

1. Health M of, editor. No 26 - Food act [Internet]. No 26. 1980. Available from: http://www.health.gov.lk/enWeb/FOODWEB/files/regulations.html

2. Democratic Socialist Republic of Sri Lanka. Food (Hygiene) Regulation: No 1742/26, the Gazette of part I General Government Notifications [Internet]. 2012. Available from: https://eohfs.health.gov.lk/food/images/pdf/regulations/food_hygiene_regulations_2011_en.pdf

3. CDPH. Good Manufacturing Practices Checklist.

4. Retail & Food Service HACCP | FDA [Internet]. [cited 2023 Sep 8]. Available from: https://www.fda.gov/food/hazard-analysis-critical-control-point-haccp/retail-food-service-haccp

5. Government of Canada. A Guide to Health Canada Inspections - Canada.ca [Internet]. [cited 2023 Sep 8]. Available from: https://www.canada.ca/en/health-canada/corporate/mandate/regulatory-role/what-health-canada-does-as-regulator/guide-inspections.html

6. WHO. Essential safety Requirements for Street-Vended Foods (Revised Edition) Food Safety Unit Division of Food and Nutrition World Health Organization [Internet]. [cited 2023 Jun 22]. Available from: https://apps.who.int/iris/bitstream/handle/10665/63265/WHO_FNU_FOS_96.7.pdf?sequence=1&isAllowed=y

7. Cruz MA, Katz DJ, Suarez JA. An assessment of the ability of routine restaurant inspections to predict food-borne outbreaks in Miami-Dade County, Florida. Am J Public Health. 2001;91(5):821.

*Supplementary file 1*
