# Supplementary material for: Effects of health educational and participatory consumer group interventions in improving food handling practices in regional director of health services area Kalutara, Sri Lanka: non-randomized controlled community trial
Source: BMC Public Health. 2024 Apr 6;24:972. doi: 10.1186/s12889-024-18481-2 (PMC10998395; doi:10.1186/s12889-024-18481-2)
Supplement: Supplementary file 2 — Supplementary Material 2. [file 12889_2024_18481_MOESM2_ESM.doc]

| **Food Establishments’ Assessment tool** | | Department of Public Health  ………………………………………….. |
| --- | --- | --- |
| Name of the Establishment | Address | |
| Type of Establishment | | Hotel | Bakery | Restaurant | Café / Juice bar/ snack bar / | Others | | --- | --- | --- | --- | --- | | |
| Inspected By:  …………………………………………………………….. | Date Time  ……………………….. ………………………………… | |
| **Use marks 5 for The Best and 1 for The Worst or if it is not relevant to the establishment, mark** *(Tick the appropriate column)* **in NR column** | | |
| Use this checklist to determine areas in the operation requiring corrective action.  Record observations, corrective action taken and keep completed records in a file for future reference. Use marks 5 for best and towards 1 for worst. | | |
|  | | |
| | **Items to be consider during inspections of establishments** | **Marks** | **NR** | **Comments / Corrective action** | | --- | --- | --- | --- | | 1. Location of the establishment |  |  |  | | 1. Maintained in good condition |  |  |  | | 1. Adequate working space |  |  |  | | 1. Designed to prevent the entrance and harbouring of pests and contaminants |  |  |  | | 1. Effective means provided to prevent cross-contamination |  |  |  | | 1. Facilitate supervision of hygienic operations |  |  |  | | 1. Designed to permit easy and proper cleaning |  |  |  | | 1. Adequate facilities are made available for food 2. heating |  |  |  | | 1. cooling (A/C, exhauster fans) | | 1. cooking | | 1. refrigerating and freezing | | 1. Sound construction |  |  |  | |  |  |  |  | | **Processing areas** |  |  |  | | 1. Floors of water-proof, non absorbent, washable and non-slippery materials |  |  |  | | 1. Walls of water-proof, non-absorbent and washable materials |  |  |  | | 1. Walls up to the operation height are smooth and without crevices and easy to cleaned |  |  |  | | 1. Working surfaces are in sound condition and maintained |  |  |  | | 1. Ceilings are constructed to prevent:  - accumulation of dirt |  |  |  | | - development of mould and flaking |  |  |  | | - and easy to clean |  |  |  | | 1. Doors fitted with smooth and non-absorbent surfaces |  |  |  | | 1. Windows are easy to clean and constructed to minimize the buildup of dirt |  |  |  | | 1. Windows fitted with cleanable insect-proof screens or mesh |  |  |  | | 1. Fans and exhaust hood are clean |  |  |  | | 1. Adequate drains provided |  |  |  | | 1. Drains constructed to avoid stagnation and back-flow of effluent |  |  |  | | 1. All surfaces and floors are clean |  |  |  | | 1. Uncooked vegetables are kept in well-ventilated, adequately-lit, rodent-proof compartments suitable for the purpose or in a refrigerator or cold room |  |  |  | | 1. Unnecessary items are not kept in processing areas. |  |  |  | | **Display and serving** |  |  |  | | 1. Cooked meat and fish and cooked products containing meat or fish which are ready to eat are stored in enclosed containers without exposure to flies and contaminates |  |  |  | | 1. Foods set out for selection by customers, are placed in proper manner |  |  |  | | 1. Tongs, forks, spoons, spatulas provided for use by workers |  |  |  | | 1. Not carrying spoon, knife or fork in the pocket of any garment or apron. |  |  |  | | 1. Wiping utensils with soiled cloths/towels that are suitable for the purpose of wiping |  |  |  | | 1. Place, carry or store foods in such a manner that not lead to contamination |  |  |  | | 1. All readily-perishable foods that have been cooked are stored in proper manner |  |  |  | | 1. Water jugs on tables or counters shall be provided with suitable covers to protect the contents from contamination |  |  |  | | 1. Ready to eat fruits and vegetables, before service to customers, are washed and thoroughly cleaned |  |  |  | |  |  |  |  | | **Overhead structures and fittings** |  |  |  | | 1. installed to avoid contamination |  |  |  | | 1. shall not hamper cleaning operations |  |  |  | | 1. designed and finished as to prevent the accumulation of dirt, development of mould and flaking |  |  |  | | **Water supply and storage** |  |  |  | | 1. Provided with adequate supply of potable water |  |  |  | | 1. Water stored in clean containers, free of hazardous substances and contaminants |  |  |  | | **Equipment and utensils** |  |  |  | | 1. Made of materials which do not transmit any toxic substances, odour or taste |  |  |  | | 1. Made of non-absorbent material resistant to corrosion and capable of withstanding repeated cleaning and disinfection |  |  |  | | 1. Designed and constructed as to prevent hygienic hazards and permit easy and thorough cleaning and disinfection |  |  |  | | 1. Containers for collection of inedible materials and waste are leak-proof and constructed of metal or other impervious materials |  |  |  | | 1. Waste collection containers are identifiable |  |  |  | | 1. Refrigerators and freezers are maintained in good repair and are in order |  |  |  | | **Other facilities** |  |  |  | | 1. Toilets conforming to sanitary requirements are provided |  |  |  | | 1. Toilets not opening directly onto food handling areas |  |  |  | | 1. Hand washing facilities and hand cleaning preparation are provided adjacent to the toilet |  |  |  | | 1. Processing areas are provided with hand washing and drying facilities. |  |  |  | | 1. Adequate and appropriate lighting is provided throughout the establishment. |  |  |  | | 1. Adequate ventilation is provided to prevent excessive build up of heat, steam condensation and dust |  |  |  | | 1. Pesticides and chemicals are stored in cabinets away from foods |  |  |  | | 1. Food wrapping materials are stored in a proper manner |  |  |  | | 1. Animal, bird or any other pets are not kept in the food establishment |  |  |  | | 1. Food handling areas are free of pests & insects |  |  |  | | 1. Waste materials are not allowed to accumulate in food handling areas. |  |  |  | | 1. All food items are kept 1.5 feet above ground |  |  |  | | 1. Not using printed papers for covering, wiping or keeping food. |  |  |  | | **Medical examination** |  |  |  | | 1. Persons working as food handlers are certified fit by Medical Officer |  |  |  | | 1. Medical examination of food handlers is carried out periodically |  |  |  | | 1. Records of Medical examinations are maintained properly and available for inspection |  |  |  | | **Personal Hygiene of food handlers** |  |  |  | | 1. Employees maintain a high degree of personal cleanliness |  |  |  | | 1. Wear suitable clean clothing |  |  |  | | 1. Food handlers do not wipe hand on his clothing |  |  |  | | 1. Workers use clean towel to wipe hands |  |  |  | | 1. Cloths used for wiping table tops are clean and changed |  |  |  | | 1. Fingernails are short, unpolished, and clean |  |  |  | | 1. Masks, caps and gloves are used appropriately |  |  |  | | 1. Not doing Betel-chewing in unhygienic way |  |  |  | | **Precautionary measures taken** |  |  |  | | 1. Foods are not placed in any place where there is a risk of contamination |  |  |  | | 1. Food handling areas are not used as a sleeping place |  |  |  | | 1. Meat and fish are stored when not being processed, at a temperature below 2^0^ C |  |  |  | | 1. Milk is kept during the whole period of storage in a refrigerator at a temperature not exceeding 7^0^ C ; |  |  |  | | 1. Bones, fish offal, and waste matter are not allowed to accumulate |  |  |  | | 1. Bones, fish offal, and waste matter Disposed of in such a manner as to prevent a nuisance being caused (Avoid smell) |  |  |  | | **Responsibilities of owners of food establishment** |  |  |  | | 1. Ensure that food handlers are supervised and instructed and trained in food hygiene matters commensurate with their activities |  |  |  | | 1. The FIFO (First In, First Out) method of   Inventory is being practiced |  |  |  | |  |  |  |  | | | |
|  | | |

*Supplementary file 2*
