# Supplementary material for: Effects of health educational and participatory consumer group interventions in improving food handling practices in regional director of health services area Kalutara, Sri Lanka: non-randomized controlled community trial
Source: BMC Public Health. 2024 Apr 6;24:972. doi: 10.1186/s12889-024-18481-2 (PMC10998395; doi:10.1186/s12889-024-18481-2)
Supplement: Supplementary file 3 — Supplementary Material 3. [file 12889_2024_18481_MOESM3_ESM.doc]

| **SELF-INSPECTION CHECKLIST** | **SAFE FOOD SAVES LIVES PROGRAMME** |
| --- | --- |
| Name of the Establishment | Address |
| Type of Establishment | | Hotel | Bakery | Restaurant | Manufacturing plant | | --- | --- | --- | --- | |
| Head Self-Inspection Team:  …………………………………………………………….. | Date Time  ……………………….. ………………………………… |
| **S = SATISFACTORY NI = NEEDS IMPROVEMENT U = UNSATISFACTORY**   - *(Tick the appropriate column)* | |
| Use this checklist to determine areas in the operation requiring corrective action.  Record observations, corrective action taken and keep completed records in a file for future reference. | |
|  | |
| | **Items to be considered during inspections** | **S** | **NI** | **U** | **Comments / Corrective action** | | --- | --- | --- | --- | --- | | 1. **General condition of the establishment** |  |  |  |  | | 1. Location of the establishment (in an area free from excessive odors and fumes) , |  |  |  |  | | 1. Sound construction (Floor cemented or tiled, with ceilings, walls, doors and windows colour washed) |  |  |  |  | | 1. Maintained in good condition (Frequent cleaning, prevent accumulation of dirt and filth, rutting washing, mopping and disinfecting) |  |  |  |  | | 1. Adequate working space (Equipment properly placed, free from unwanted items and rubbish) |  |  |  |  | | 1. Adequate light and ventilation is provided throughout the establishment |  |  |  |  | | 1. **Serving and processing areas** |  |  |  |  | | 1. All surfaces and floors kept in a neat and tidy manner. (Frequent cleaning) |  |  |  |  | | 1. Tables, racks, cupboards etc.., kept in a neat and tidy manner. |  |  |  |  | | 1. All food items kept cleanly and orderly without exposing to any contamination. (from dust, dirt or filth, flies and insects) |  |  |  |  | | 1. Provided with adequate supply of water and soap for hand washing |  |  |  |  | | 1. Water jugs on tables or counters are provided with suitable covers to protect the contents from contamination |  |  |  |  | | 1. **Personal Hygiene** |  |  |  |  | | 1. Employees maintain a high degree of personal cleanliness. |  |  |  |  | | 1. Wear suitable clean clothing |  |  |  |  | | 1. Cloths used for wiping table tops are clean and changed |  |  |  |  | | 1. Manner of handling of food by workers satisfactory. |  |  |  |  | | 1. Tongs, forks, spoons, spatulas provided and used by workers |  |  |  |  | | 1. **Other facilities** |  |  |  |  | | 1. Containers for collection of waste are provided with protective covers. |  |  |  |  | | 1. Toilets provided in a satisfactory manner with adequate water supply |  |  |  |  | | 1. Hand washing facilities and hand cleaning preparation are provided adjacent to the toilet |  |  |  |  | | 1. Animal, bird or any other pets are not kept in the food establishment |  |  |  |  | | 1. Waste materials are not allowed to accumulate in food handling areas. |  |  |  |  | | 1. **Responsibilities of owners’ of food establishment** |  |  |  |  | | 1. Ensure that food handlers are supervised and instructed and trained in food hygiene matters commensurate with their activities |  |  |  |  | | 1. Employees have necessary knowledge of food hygiene principles and practices |  |  |  |  | | 1. The FIFO (First In, First Out) method of Inventory is being practiced |  |  |  |  | | 1. Ensure maintaining establishment in a high standard conforming to all legal requirements |  |  |  |  | | |
|  | |

**Grading system**

Each tick of “S” has 4 marks.

**Grading scheme for “S” marks (%)**

1-25 (Poor) 26- 50 (Unsatisfactory) 51-75 (Satisfactory) 76-100 (Good)

NOTE- “NI” is used only for monitoring purposes

The overall grading of the establishment:

(State as POOR / UNSATISFACTORY / SATISFACTORY / GOOD)

Comments of the Self-Inspection Team

………………………………………………………………………………………………………………………………………………………………………………………………………………………………………………………………………………………………………………………………………………………………

Signature: …………………………………….. Date: ………………………….

*Supplementary file 3*
