# Supplementary material for: Effects of health educational and participatory consumer group interventions in improving food handling practices in regional director of health services area Kalutara, Sri Lanka: non-randomized controlled community trial
Source: BMC Public Health. 2024 Apr 6;24:972. doi: 10.1186/s12889-024-18481-2 (PMC10998395; doi:10.1186/s12889-024-18481-2)
Supplement: Supplementary file 4 — Supplementary Material 4. [file 12889_2024_18481_MOESM4_ESM.docx]

Supplementary Table 1: Distribution of Percentage Providing Correct Answers Related to Methods of Transmission for Foodborne Diseases among Food Handlers

| **Description** | **No:** | **%** |
| --- | --- | --- |
| Knowledge on methods of transmission for food-borne diseases |  |  |
| (Correct responses are 1,3,5 and 6, incorrect responses are 2.4,7) |  |  |
| 1.Through drinking unsafe water | 49 | 5.4 |
| 2.Air transmission | 16 | 1.8 |
| 3.By consuming unsafe, infected food | 49 | 5.4 |
| 4.Mosquitoes | 9 | 1 |
| 5.By handling of food-by-food handlers infected with food-borne diseases | 18 | 2 |
| 6.Answers 1,3 and 5 are correct | 666 | 73.7 |
| 7.Don’t know | 65 | 7.2 |
| 8.Missing | 32 | 3.5 |
| Knowledge about allowing to operate in food premises to a person who is infected or suspected of suffering from food-borne diseases |  |  |
| 1.Person can be allowed to operate in a job other than food handling | 4 | 4 |
| 2.Person cannot be allowed to operate in any job | 855 | 94.6 |
| 3. Person can be allowed to operate in any job | 15 | 1.7 |
| 4. Don’t know | 0 | 0 |
| 5.Missing | 30 | 3.3 |
|  |  |  |
| Knowledge on correct temperature, at which milk should be stored at |  |  |
| 1.Above 2^o^C | 39 | 4.3 |
| 2. Less than 2^o^C | 20 | 2.2 |
| 3.Stable, continuous temperature at 7^o^C for short period of time | 2 | 0.4 |
| 4.Stable, continuous temperature at 7^o^C for whole period of storing time | 8 | 0.7 |
| 5. Don’t know | 796 | 88.1 |
| 6.Missing | 39 | 4.3 |
|  |  |  |
| Knowledge on correct temperature, in which fish or meat should be stored at |  |  |
| 1.Less than +2^o^C | 54 | 6 |
| 2.Less than +0^o^C | 42 | 4.6 |
| 3.Less than +8^o^C | 16 | 1.8 |
| 4.Less than -10^o^C | 23 | 2.5 |
| 5.Don’t know | 737 | 81.5 |
| 6.Missing | 32 | 3.5 |
| **Total** | **904** | **100** |

Almost 75% of the study sample reported the correct answer, which was the 6^th^ stem for the question about the methods of transmission of foodborne diseases, whereas assessing knowledge on the stem of “allowing an infected person of foodborne disease to operate in food premises” was reported correct answer by 95% of the sample. However, an assessment of knowledge on storing temperature of fish or meat and milk showed a clear majority of more than 90% of food handlers unaware of the correct answer.

Supplementary Table 2: Distribution of Knowledge on Food Handling among Food Handlers after Summed Particular Set of Questions as Individual Domains

|  | **Knowledge Domain*** | | | | | |
| --- | --- | --- | --- | --- | --- | --- |
|  | General Knowledge | Activities refrained at food establishment | Activities adopted at the time serving food | Knowledge on food-borne diseases | Consequences due to food-borne diseases | **Overall knowledge score** |
| No of questions | 8 | 10 | 8 | 7 | 5 | 38 |
| Total marks | 16 | 20 | 16 | 14 | 10 | 76 |
| Median | 16 | 16 | 16 | 10 | 10 | 14 |
| Mode | 16 | 17 | 16 | 10 | 10 | 15 |

*answers given for a set of questions in a single domain were summed and recoded to have a final score for a particular domain among food handlers

The median domain scores varied from 10 of 10 total scores for the knowledge of consequences due to foodborne diseases to 16 of 20 total scores for the knowledge of activities refrained at the food establishment domain.

Supplementary Table 3: Distribution of Knowledge on Storing Milk, Meat, Fish and Fast-food Items Containing Fish and Meat and Waste Disposal among Food Handlers after Summed as Individual Domain

|  | **Knowledge Domain*** | |
| --- | --- | --- |
|  | General Knowledge on storing milk, meat, fish and fast-food items containing fish and meat | Knowledge on activities adopted by disposing waste matter like kitchen waste, fish offal, bones etc |
| No: of questions | 3 | 5 |
| Total marks | 6 | 10 |
| Median | 0 | 8 |
| Mode | 0 | 8 |

*calculated by the sum of answers given for a set of questions in a particular domain

Supplementary Table 4: Distribution of Knowledge on Storing milk, Meat, Fish and Fast-food Items Containing Fish and Meat and Waste Disposal among Food Handlers according to Level of Knowledge

|  | **Knowledge Domain** | | | |
| --- | --- | --- | --- | --- |
|  | General Knowledge on storing milk, meat, fish and fast-food items containing fish and meat | | Knowledge on activities adopted by disposing waste matter like kitchen waste, fish offal, bones, etc | |
|  | No: | % | No: | % |
| Very poor | 873 | 96.6 | 61 | 6.7 |
| Poor | 4 | 0.4 | 176 | 19.5 |
| Satisfactory | 20 | 2.2 | 63 | 7 |
| Good | 7 | 0.8 | 604 | 66.8 |
| Total | 904 | 100 | 904 | 100 |
|  |  | |  | |

*Supplementary file 4*
